# Regarding the parallel opposed editorial about: 3D printing technology will eventually eliminate the need of purchasing commercial phantoms for clinical medical physics QA procedures

**DOI:** 10.1002/acm2.12448

**Published:** 2018-09-06

**Authors:** Marius Treutwein

**Affiliations:** ^1^ Department of Radiotherapy Regensburg University Medical Center Regensburg Germany

Dr. Ehler and Dr. Craft discuss not only the topic in the narrow sense but also illustrate the broad spectrum of applications of 3D printing in medical physics in radiotherapy departments.^1^ Both researchers illustrate that low cost 3D printers will generally not fulfill the requirements for all types of phantoms and accessory in medical physics. High quality printers on the other hand might be too expensive for single phantoms for small and nonacademic clinics.

Dr. Ehler has pointed out that 3D printing is not restricted to production of phantoms for radiotherapy. Pourabdollhian and Copani^2^ discussed that increasing production of customized medical products can extend the business of a clinic to a healthcare product manufacturer. Such a clinic would have its own engineering institute with knowhow and enough equipment also in the case of breakdown and thus avoid some of the problems described by both opponents. However, 3D printing needs not to be performed in the neighborhood. The process files can be sent electronically to a service contractor. The responsibility for air voids, as mentioned by Dr. Ehler, or other defects, keeping of density prescriptions and measures can be delegated by contract, as well as jamming of the printer as described by Dr. Craft. In an economic sense such phantoms are also “commercial” as we have to pay for them but we get influence in the design. Maybe, the manufacturers of our well‐known commercial equipment will offer such services. They know the requirements of their customers very well.

It has been mentioned in the introduction that 3D printing is not a new concept. Therefore, an increasing number of 3D printing services can be found on the web. In earlier years we could order shielding blocks, now we will get phantoms and accessory. A recent publication in this journal^3^ demonstrates us the production of electron apertures with low cost 3D printers. In my opinion, we will have such low cost printers for daily accessory production. We will have the knowhow (or have it yet) to create the process files and if our low cost printer cannot print our product, we will order it. In this way we will not eliminate but reduce the need of purchasing commercial phantoms.
